# Shear bond strength of a flash-free orthodontic adhesive system after thermal aging procedure

**DOI:** 10.4317/jced.532744

**Published:** 2019-08-01

**Authors:** Carlos González-Serrano, Eugenia Baena, María-Victoria Fuentes, Alberto Albaladejo, Manuel Míguez-Contreras, Manuel O. Lagravère, Laura Ceballos

**Affiliations:** 1 PhD Student, Area of Stomatology, Health Sciences Faculty, Rey Juan Carlos University, Alcorcón, Madrid, Spain; 2 Assistant Professor, Area of Stomatology, Health Sciences Faculty, Rey Juan Carlos University, Alcorcón, Madrid, Spain; 3 Associate Professor, Department of Surgery, Faculty of Medicine, University of Salamanca, Salamanca, Spain; 4 Assistant Professor, Department of Stomatology, Health Sciences Faculty, Alfonso X el Sabio, Madrid, Spain; 5 Associate Professor, School of Dentistry, Faculty of Medicine & Dentistry, University of Alberta, Edmonton, Canada; 6 Full Professor, Area of Stomatology, Health Sciences Faculty, Rey Juan Carlos University, Alcorcón, Madrid, Spain

## Abstract

In this article by Carlos and colleagues (J Clin Exp Dent. 2019 Feb 1;11(2):e154-61), there is an error in the Material and Methods of the abstract. The correct Material and Methods of the abstract is: Material and Methods: A total of 120 human premolars were randomly divided into two groups (n=60) according to the orthodontic adhesive used: APC Flash-Free Adhesive Coated Appliance System (APC FF) or Transbond PLUS Color Change Adhesive (TP), as control. A SBS test was performed and ARI value for each specimen was also assessed. Results were analyzed by two-way ANOVA and Tukey's Chi-square test (*p*<0.05).

